# Mosquitocidal toxin-like islands in *Bacillus thuringiensis* S2160-1 revealed by complete-genome sequence and MS proteomic analysis

**DOI:** 10.1038/s41598-024-66048-3

**Published:** 2024-07-02

**Authors:** Yan Zhou, Wenfei Zhang, Yusong Wan, Wujun Jin, Yan Zhang, Youzhi Li, Baoshan Chen, Mingguo Jiang, Xuanjun Fang

**Affiliations:** 1 Guangxi Key Laboratory for Polysaccharide Materials and Modifications, School of Marine Sciences and Biotechnology, Guangxi Minzu University, Nanning, 530006 China; 2 Hainan Institute of Tropical Agricultural Resources, Sanya, 572025 Hainan China; 3 Institute of Life Science, Jiyang College of Zhejiang A&F University, Zhuji, 311800 Zhejiang China; 4 Ministry of Education Key Laboratory for Ecology of Tropical Islands, College of Life Sciences, Hainan Normal University, Haikou, 571158 Hainan China; 5 Biotechnology Research Institute, Chinese Academy of Agricultural Sciences, Beijing, 100081 China; 6 School of Life Science and Technology, Harbin Institute of Technology, Harbin, 150001 China; 7 Guangxi Research Center for Microbial and Enzyme Engineering Technology, College of Life Science and Technology, Guangxi University, Nanning, 530004 Guangxi China; 8 State Key Laboratory for Conservation and Utilization of Subtropical Agro-bioresources, College of Agriculture, Guangxi University, Nanning, 530004 Guangxi China

**Keywords:** Mosquitocidal toxin-like island, *Bacillus thuringiensis*, Bt S2160-1, Complete-genome sequence, LTQ-Orbitrap LC–MS/MS, Biological techniques, Microbiology

## Abstract

Here, we present the whole genome sequence of Bt S2160-1, a potential alternative to the mosquitocidal model strain, *Bti*. One chromosome genome and four mega-plasmids were contained in Bt S2160-1, and 13 predicted genes encoding predicted insecticidal crystal proteins were identified clustered on one plasmid pS2160-1p2 containing two pathogenic islands (PAIs) designed as PAI-1 (Cry54Ba, Cry30Ea4, Cry69Aa-like, Cry50Ba2-like, Cry4Ca1-like, Cry30Ga2, Cry71Aa-like, Cry72Aa-like, Cry70Aa-like, Cyt1Da2-like and Vpb4C1-like) and PAI-2 (Cyt1Aa-like, and Tpp80Aa1-like). The clusters appear to represent mosquitocidal toxin islands similar to pathogenicity islands. Transcription/translation of 10 of the 13 predicted genes was confirmed by whole-proteome analysis using LTQ-Orbitrap LC–MS/MS. In summary, the present study identified the existence of a mosquitocidal toxin island in *Bacillus thuringiensis*, and provides important genomic information for understanding the insecticidal mechanism of *B. thuringiensis*.

## Introduction

*Bacillus thuringiensis* (Bt) is a Gram-positive, spore-forming soil bacterium, which has been isolated from many different natural habitats. Bt is characterized by the presence of parasporal crystal proteins (ICPs) during sporulation that have insecticidal activity against the larvae of specific insect species, as well as invertebrate pests^1,2^. For this reason, Bt has been broadly used as a biological pesticide for the management of several agricultural and forestry pests, as well as mosquitoes.

*Bacillus thuringiensis* subsp. *israelensis* (*Bti*) was the first subspecies of Bt found to exhibit Diptera-larvicidal activity. This mosquitocidal bacterium was commercially developed into an effective, environmental-friendly, biological control agent for managing the vectors of mosquito-borne diseases^3,4^. The *Bti* native isolate harbors a single circular replicon of approximately 16 kb and eight circular plasmids ranging from 5 to 210 kb in size^5,6^. Four major ICPs (Cry4Aa, Cry4Ba, Cry10Aa, and Cry11Aa with sizes of 134, 128, 72, and 27 kDa, respectively) have been identified in *Bti*, all of which are located on a pBtoxin mega-plasmid (128 kb)^7–9^.

Since the original identification of *Bti*, many new mosquitocidal strains have been discovered and the Bt mosquitocidal protein database has been greatly enriched, relative to the first six mosquitocidal proteins of *Bti* (Cry4Aa, Cry4Ba, Cry10Aa, Cry11Aa, Cyt1Aa and Cyt2Ba). Cry11Ba1 and Cry11Bb1 proteins were identified in the *B. thuringiensis* subsp. *jegathesan* and *B. thuringiensis* subsp. *medellin*, respectively, and exhibited 10 times greater mosquitocidal activity than the strongest toxin activity in the *Bti* strain^10,11^. Subsequently, Cry19Aa and ORF2 were identified in the *B. thuringiensis* subsp. *jegathesan* with specific toxicity against *Anopheles stephensi* and *Culex pipiens*, two species of mosquito. Notably, stronger toxicity was observed when Cry19A and ORF2 were combined than when either protein was used alone, however, neither protein is toxic to *Aedes aegypti*^12,13^. Cry19Ba shares 49% amino acid identity with Cry19Aa from the *B. thuringiensis* subsp. *jegathesan*, and also exhibits mosquitocidal activity against *C. pipiens*^14^ and *C. pipiens molestus*^15^.

In addition to the *B. thuringiensis* subsp. *jegathesan* toxin proteins mentioned above, this subspecies also produces Cry60Aa and Cry60Ba mosquitocidal toxins, both of which are toxic to *Culex quinquefasciatus*, either individually or in combination^16^. Cry27Aa protein in the *B. thuringiensis* serovar *higo* has a highly-specific larvicidal toxicity against *A. stephensi*, but no toxicity against *C. pipiens molestus* or *A. aegypti*^17^. Cry20Aa protein from the *B. thuringiensis* subsp. *fukuokaensis* has a highly different amino acid sequence, except for conserved regions, than other mosquitocidal toxins. Cry20Aa exhibits low larvicidal activity against *A. aegypti* and *C. quinquefasciatus*, which may be due to the rapid proteolysis of this toxin^18,19^. Cry44Aa ICP produced by the *B. thuringiensis* subsp. *entomocidus* INA288 is highly toxic to the second-instar of *C. pipiens pallens* and *A. aegypti*^20,21^. Cry29A and Cry30A proteins have no toxicity against mosquito larvae individually, however, they act synergistically when combined and exhibit a high level of toxicity to *A. aegypti*^22^. Besides, Cry80Aa1 protein was reported that exhibited toxicity to third instar larvae of *C. pipiens pallens* in 2020^23^. A number of other novel mosquitocidal toxins have also been identified, including some belonging to the Mtx and Bin toxin families^24^, such as, Epp containing Cry-ETX/MTX conserved domains from strain S3580-1 is toxic to mosquitoes and prodenia litura larvae^25^.

The mosquitocidal isolate, *B. thuringiensis* S2160-1, was originally isolated from soil samples collected in the Dawangling Forest Nature Reserve (Guangxi, China) and has been maintained in culture by the Hainan Institute of Tropical Agricultural Resources (HITAR). The Bt S2160-1 isolate has been identified as a potential alternative to *Bti*, due to its high mosquitocidal activity^26^. The strain has been officially registered and preserved in the China General Microbiological Culture Collection Center (Collection No 13274). Bt S2160-1 has ivory white, flattened, colony morphology with a matte appearance on LB plates with irregularly serrated margins. A high number of spherical parasporal crystals can be observed during the sporulation of Bt S2160-1 using scanning electron microscopy.

Zhang et al.^26^ successfully cloned four novel toxin genes, *cry50Ba1*, *cry54Ba1*, *cry30Ea1*, and *cry30Ga2* from Bt S2160-1 and conducted a MALDI-TOF MS analysis of highly-expressed protein bands extracted from gels. Results revealed that *cry50Ba1* and *cry54Ba1* genes were expressed in vivo, while *cry30Ea1* and *cry30Ga2* genes were either expressed at a low level or not detected. An analysis of in vitro expression further confirmed that Cry50Ba1 and Cry54Ba1 expression can be induced while Cry30Ea1 and Cry30Ga2 expression were not inducible. Bioassay of the Cry50Ba1 and Cry54Ba1 ICPs indicated that Cry50Ba1 had no mosquitocidal activity (LC_50_ > 2000 μg/mL), and that Cry54Ba1 exhibited a very low level of mosquito toxicity^26^. Subsequently, Zhang et al. identified a 130 kDa toxin named Cry4Cb3 using MALDI–TOF/TOF mass spectrometry, which had mosquitocidal activity against *C. quinquefasciatus* (LC_50_: 22.49 μg/mL)^27^. Zhang’s independent research indicated that Cry50Ba, produced by Bt S2160-1, is a dominant toxin, exhibiting strong mosquitocidal activity against *C. quinquefasciatus* (LC_50_: 73.87 ng/mL, 95% FL 51.40–102.75 ng/mL)^28^. Notably, however, the toxicity of the individual expressed proteins encoded by the cloned genes was far lower than a whole crude protein mixture of S2160-1 (LC_50_: 5.668 ng/mL) against *C. quinquefasciatus* larvae^26^.

Based on the experimental data reported by our team, we speculate that maybe other toxic proteins are undiscovered in Bt S2160-1 strain, which may exhibit highly mosquitocidal activity or they may act synergistically together to produce an unexpected, high-level of mosquitocidal activity. In addition, we hypothesized that the mosquitocidal toxins might cluster on plasmids and form mosquitocidal toxin islands similar to pathogenic islands (PAIs).

Therefore, to identify all the toxins in Bt S2160-1, the complete-genome sequence of Bt S2160-1 was carried out in current study. The obtained genome sequence was then analyzed to produce the predicted structure of the chromosome and plasmid genomes, as well as the genomic sequence of the toxin-encoding genes. LTQ Orbitrap MS analysis of the proteome of Bt S2160-1 was conducted to validate the expression of the predicted toxin proteins at the whole-proteome level. The combined approaches described above were used to determine the existence of a mosquitocidal toxin island in Bt S2160-1.

## Materials and methods

### Strain and medium

*Bacillus thuringiensis* S2160-1 was isolated by Zhang et al. and cultured as it mentioned^26^. A culture of the isolate has been maintained by the Hainan Institute of Tropical Agricultural Resources (HITAR). The strain is registered and deposited in the China General Microbiological Culture Collection Center (Collection code: CGMCC No 13274).

### Complete-genome sequence and assembly

Genomic DNA of Bt S2160-1 was extracted using an E.Z.N.A.® Bacterial DNA Kit (OMEGA, Cat.D3350) according to the manufacturer’s instructions. Complete-genome sequencing of the whole-genome of Bt S2160-1 strain was performed the MGISEQ-2000 platform and Oxford Nanopore Technologies (ONT) PromethION P24 device at BGI (BGI, Shenzhen, China). High quality reads were obtained from the original data generated by the sequencing platform after removing redundant, low-quality reads, such as low mass value bases and adaptors, to ensure the accuracy of the subsequent assembly was performed using Nanopore Assembly Method (Canu). The complete-genome data of Bt S2160-1 was submitted into GenBank (Accession number NSKZ00000000).

### Prediction of ORFs and CDSs

The ORFs of the assembled genome of Bt S2160-1 were predicted using GLIMMER (http://ccb.jhu.edu/software/glimmer/index.shtml, version 3.02) software^29^, while the prediction of coding genes and their corresponding encoded protein was completed using MetaGeneMark (http://exon.gatech.edu/GeneMark/metagenome/index.cgi, version 1.0) software^30^.

A local Bt toxin database was built for BLAST using known Bt toxin protein sequences, and the new Bt toxic protein nomenclature database the Bacterial Pesticidal Protein Resource Center (https://www.bpprc-db.org/home/) to confirm the predicted toxins. A total of 1140 amino acid sequences of Bt toxin proteins (up to January 2024) were downloaded from public data resources to construct a local database for BLAST alignment analysis.

### Preparation of Bt S2160-1 total protein

Bt S2160-1 strains were streaked and cultured on LB plate medium and then a single colony was picked with a needle tip and cultured in 5 mL LB medium at 28 °C at 200 rpm for 12 h. The bacterial culture was then transferred to a conical flask containing 300 mL Nutrient Broth liquid culture medium at a ratio of 1:100 and cultured at 28 °C at 200 rpm. Subsequently, 15 mL of the bacterial culture, which was cultured continuously for 72 h until spores and crystals were completely formed, were collected every 4 h. A total of 18 cultured samples were collected over the72 h culture period and stored at − 20 °C for later use.

Each collected sample was centrifuged at 4000*g* at 4 °C for 20 min to harvest bacterial cells. Then, 15 mL 1 × TE buffer was added to re-suspend the bacterial cells, and the cultures were again centrifuged at 4000*g* at 4 °C for 20 min and the resulting supernatant was discarded. The bacterial cells from all of the sampled time points were combined, washed 3 times with 1 M NaCl, and finally re-suspended in 15 mL protein lysate buffer (50 mM Tris–HCl, 500 mM NaCl, pH 8.0).

Total proteins of Bt S2160-1 were extracted using an ultrasonic cell crusher (SONICS VCX750, SONICS & MATERIALS, INC. 53, Church Hill Rd. Newtown, CT USA), using the working conditions: 25% Ampl, pulse on 3 Sec., pulse off 15 Sec., working time 20 min. A 5 μL extracted protein solution was taken to determine the quality of the extracted proteins by SDS-PAGE electrophoresis, and 1% PMSF (100 mM), was added to each of the remaining protein solutions, which were then stored at − 80 °C for later use.

### The whole protein expression profile of Bt S2160-1 identified by LTQ-Orbitrap nano-LC–MS/MS

Bt S2160-1 was cultured at 28 °C, and culture samples were collected once every 4 h, for a total of 18 time points over three consecutive days. Proteins from each sample were extracted by sonication (SONICS VCX750, SONICS&MATERIALS, INC. 53 Church Hill Rd. Newtown, CT USA). Total protein mixtures were obtained by mixing the extracted proteins from the 18 sampled time points together and this mixture was used for analyzing the protein expression profile of Bt S2160-1. The time points covered the complete growth cycle from the vegetative phase to the end of the sporulation phase. The protein samples were digested overnight in an incubator at 37 °C by adding trypsin (trypsin: protein = 1:40) and analyzed by SDS-PAGE electrophoresis for complete digestion.

The enzymatically digested protein samples were dried using a low temperature vacuum concentrator and then re-dissolved in SCX solvent A (10 mmol/L potassium phosphate buffer in 20% ACN). The samples were separated by a strong cation exchanger (SCX) with a PolySulfoethyl A column (PolyLC, Columbia, MD; 200 Å, 5 μm, 200 × 2.1 mm). A flow rate for the SCX solvent A was set at 0.3 mL/min, and the gradient concentration of solvent B (10 mM KH_2_PO_4_, 500 mM KCl, 20% acetonitrile) was set as follows: 0% of solvent B for 0–5 min, 0–40% of solvent B for 5–50 min, 40–100% of solvent B for 50–55 min, 100% of solvent B for 55–63 min and 100–0% of solvent B for 63–70 min. Separate fractions were collected and individually lyophilized. Subsequently, 10 μL ddH_2_O was added to dissolve each fraction. The dissolved fractions were then combined into 8 fractions, which were fully mixed and prepared for desalination.

LTQ-Orbitrap Nano LC–MS/MS analysis of all 8 peptide samples was performed using an LTQ-Orbitrap nano-LC–MS/MS system (LTQ-Orbitrap Elite Mass Spectrometer, Thermo Fisher Scientific, Bremen, Germany) according to the manufacturer's instruction. The operating parameters of the mass spectrometer were as follows: The voltage of electrospray was set as 1.5 kV; the primary MS spectrum was detected by Orbitrap scanning in the mass scanning range of the m/z range 350–2000 with MS resolution of 60,000; the primary MS (MS) scans were fragmentated into the secondary MS/MS spectrum by collision induced dissociation (CID) mode at a normalized collision energy of 35% with mass resolution of 15,000. The MS data were collected automatically.

The acquired MS Data were queried and matched using Proteome Discoverer version 1.3 (Thermo Scientific) with SEQUEST search engines based on the predicted protein database of Bt S2160-1 genome as determined by the whole genome sequence and the downloaded Bt toxic protein nomenclature database (http://www.btnomenclature.info/).

The database search parameters were as follows: Digested by Trypsin, and a maximum of two missing digestion sites was allowed; the fixed modification was carbamidomethylation of cysteine, while variable modifications were methylation (+ 57.021 Da) and oxidation of methionine (+ 15.995 Da), respectively; the mass deviations of the primary mass spectrometry (MS) and the secondary mass spectrometry (MS/MS) were 0.1da and 0.3 Da, respectively; the secondary MS/MS scans were matched manually in order to reduce the mismatching rate; the strict threshold of False discovery rate (FDR) was set to ≤ 1% as a cut-off value for reporting result of PSMs (Peptide Spectrum Match); the other SEQUEST parameters remained at default values; the search results were manually validated.

## Results

### Complete-genome sequence analysis of Bt S2160-1

**Figure 1:**
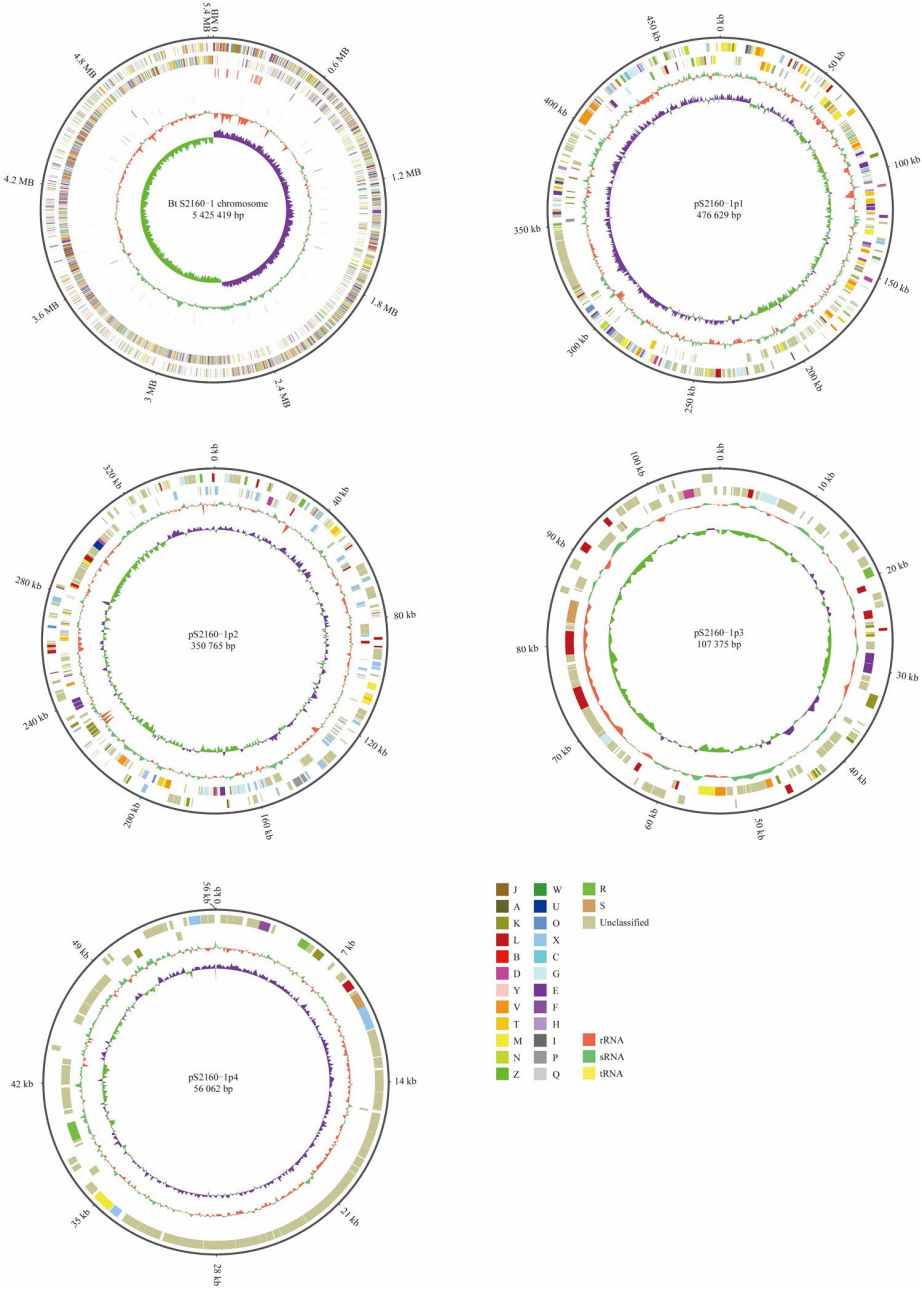
Visual map of the complete genome of Bt S2160-1.

Complete-genome sequencing of Bt S2160-1 strain (GenBank accession number CP149952-CP149956) was performed on the MGISEQ-2000 platform and Oxford Nanopore Technologies (ONT) PromethION P24 device at BGI (Shenzhen, China) in 2021. Total reads of 8,764,210 were obtained which represent 2,767,081,761 bp and total 431× fold coverage of the genome. Subsequently, the whole genome comprises five replicons: one chromosome genome that is 5,425,419 bp in length with a GC content of 35.36%) and four plasmids, containing pS2160-1p1, is 476,629 bp in length with a 32.80% GC content, pS2160-1p2, is 350,765 bp in length with a 33.24% GC content, pS2160-1p3, is 107,375 bp in length with a 33.16% GC content, and pS2160-1p4 is 56,062 bp in length with a 34.47% GC content. A complete genome map of the Bt S2160-1 was also constructed using the GC viewer server tool with default parameters (Fig. 1).

MetaGeneMark revealed that a predicted total of 6553 encoding genes in the Bt S2160-1 genome, comprising 5541 CDS in the chromosome genome, 512, 416, 144 and 80 coding genes in four plasmids of pS2160-1p1, pS2160-1p2, pS2160-1p3 and pS2160-1p4, respectively (Table 1). The chromosome also contains 42 rRNAs (14 23S, 14 16S, and 14 5S), 106 tRNAs, and 39 sRNAs. DNA regions related with genetic mobility were found along the chromosome and plasmids, among these we found 14 large prophage regions and 124 insertion sequences for transposases (Tnp). With only one CRISPR in chromosome, but lack of in the four plasmids, it is in agreement with most of the Bt strains lacked a functional CRISPR system may allowed higher frequency of horizontal gene transfer (HGT) to obtain selective genetic traits for better adaptability to diverse environments^31^.

**Table 1:** Features of the genome of Bt S2160-1.

Type	Chromosome	pS2160-1p1	pS2160-1p2	pS2160-1p3	pS2160-1p4
CDS	5541	455	364	121	72
tRNA	106	–	–	–	1
5s_rRNA (Denovo)	14	–	–	–	–
16s_rRNA (Denovo)	14	–	–	–	–
23s_rRNA (Denovo)	14	–	–	–	–
sRNA	34	2	1	2	
TRF:TandemRepeat	341	36	54	7	4
Transposase	42	19	59	4	0
Prophage	9	3	1	–	1
CRISPR	1	–	–	–	–

Subsequently, the toxin-encoding genes were identified by performing a local BLASTP alignment query, combined with a search of the PFAM database, as well as the established rules of Bt toxin classification^32^. Finally, 13 predicted toxins were dug and shown in Table 2.

**Table 2:** Homology analysis of 13 predicted toxins harbored on mega-plasmids of Bt S2160-1.

ORF ID	GenBank accession no.	Matched based on the Bt toxin nomenclature’s database			
		Best match	Comments	Identity (%)	Conserved domains
pS2160-1p2_id_6063	ADV57670	Cry 54Ba2	Full Coverage	100	Endotoxin_N, Endotoxin_M, delta-Endotoxin_C
pS2160-1p2_id_6065	ACC95445	Cry30Ea4	Full coverage	100	Endotoxin_N, Endotoxin_M, delta- Endotoxin_C
pS2160-1p2_id_6074	MF974873	Cry69Aa-like	Full coverage, but 1aa different	99.92	Endotoxin_N, Endotoxin_M, delta- Endotoxin_C
pS2160-1p 2_id_6087	MF981850	Cry50Ba2-like	Full coverage, but 21aa deletion	99.82	Endotoxin_N, Endotoxin_M, delta- Endotoxin_C
pS2160-1p 2_id_6093	AHG25301	Cry4Ca1-like	Full coverage	94	Endotoxin_N, Endotoxin_M, delta- Endotoxin_C
pS2160-1p2_id_6113	ADW27188	Cry 30Ga2	Full coverage, but 22aa deletion	100	Endotoxin_N, Endotoxin_M, delta- Endotoxin_C
pS2160-1p2_id_6137	MG932952	Cry71Aa-like	Full coverage, but 19aa deletion	99.00	Endotoxin_N, Endotoxin_M, delta- Endotoxin_C
pS2160-1p2_id_6145	MF974872	Cry72Aa-like	Full coverage, but 19aa deletion	38	Endotoxin_N, Endotoxin_M, delta- Endotoxin_C
pS2160-1p2_id_6159	MF981849	Cry70Aa-like	Full coverage	72.04	Endotoxin_N, delta- Endotoxin_C
pS2160-1p2_id_6167	MF981848	Cyt1Da1-like	No comment	20.60	PI-PLC-X + RICIN
pS2160-1p2_id_6194	AGN32390	Vpb4Ca1-like	No comment	22.8	HBL-RICIN
pS2160-1p 2_id_6259	MF981852	Cyt1Aa-like	No comment	27.85	RICIN
pS2160-1p 2_id_6293	MF981851	Tpp80Aa1-like	No comment	87.9	RICIN + Toxin_10

Best match done based on the bacterial pesticidal protein database.

*Homologous gene.

### The mosquitocidal toxin island on the plasmid pS2160-1p2 of Bt S2160-1

**Figure 2:**
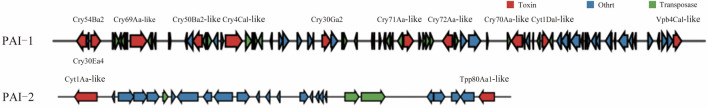
Representation of PAIs. Pesticidal proteins are represented in red arrows, transposases in green arrows and other ORF into the PAIs with blue arrows.

According to the sequences analysis, the mega-plasmid pS2160-1p2 of Bt S2160-1 harbors all the genes that encode for potential pesticidal proteins (PPs), which are grouped in PAIs, and their annotation was done according to the new database of PPs. The plasmid pS2160-1p2 contains two PAIs designed as PAI-1 and PAI-2 (Fig. 2). The length of PAI-1 harboring eleven PPs is 101.6 kbp and contains nine cry genes with 3-domain encoding for Dipteran specific Cry proteins (Cry54Ba, Cry30Ea4, Cry69Aa-like, Cry50Ba2-like, Cry4Ca1-like, Cry30Ga2, Cry71Aa-like, Cry72Aa-like, Cry70Aa-like) and two genes codifying for other insecticidal toxin classes (Cyt1Da2-like and Vpb4C1-like) (Fig. 2). The PAI-2 of 30.6 kb contains two genes encoding for PPs, name Cyt1Aa, and Tpp80Aa1. The toxin genes on these plasmids form mosquitocidal toxin islands that are similar to pathogenicity islands^33^.

### The virulence factors in Bt S2160-1 genome

Bt encodes a large number of virulence-associated genes. We searched for these genes and found that most of them were found in the chromosome of Bt S2160-1 strain (Table 3), while some virulence factors were found in the mega-plasmid pS2160-1p2, such as chitin-binding protein (cbp-2), Collagenases (colA-1 and colA-2) (Table 3).

**Table 3:** Genes coding for virulence factors in Bt S2160-1 genome.

Virulence factor	Location	Start	Stop	Strand	Virulence factor	Location	Start	Stop	Strand
Insecticidal toxins					cbp-4	Chromosome	2,703,846	2,705,105	−
Cry 54Ba	pS2160-1p2	57,402	59,501	−	cbp-5	Chromosome	2,734,983	2,735,648	−
Cry30Ea	pS2160-1p2	60,580	62,646	+	cbp-6	Chromosome	3,392,175	3,393,551	−
Cry69Aa-like	pS2160-1p2	69,305	72,955	+	Phospholipases				
Cry50Ba-like	pS2160-1p2	83,149	85,197	−	plc (phospholipase C)	Chromosome	689,723	690,574	+
Cry4Ca-like	pS2160-1p2	90,391	94,080	+	plc (phospholipase C)	Chromosome	3,390,525	3,392,003	+
Cry30Ga	pS2160-1p2	111,815	113,662	+	pldB (lysophospholipase)	Chromosome	4,690,520	4,691,365	−
Cry71Aa-like	pS2160-1p2	129,884	132,034	+	plc (phospholipase C)	pS2160-1p3	15,620	16,867	+
Cry72Aa-like	pS2160-1p2	138,538	140,673	−	plc (phospholipase C)	pS2160-1p4	36,994	37,287	+
Cyt1Da1-like	pS2160-1p2	160,636	162,135	+	Metalloproteases				
Vpb4Ca1-like	pS2160-1p2	189,730	191,505	+	inhA-1	Chromosome	683,146	685,545	+
Cyt1Aa-like	pS2160-1p2	255,582	257,219	−	inhA-2	Chromosome	1,257,715	1,260,072	+
Tpp80Aa1-like	pS2160-1p2	285,090	286,211	−	inhA-3	Chromosome	2,899,583	2,901,970	−
Chitinases					inhA-4	Chromosome	2,981,399	2,983,819	−
ChiA	Chromosome	431,185	433,251	−	Camelysin				
ChiD	Chromosome	3,665,053	3,666,135	+	cotN	Chromosome	1,256,127	1,256,720	+
Chitin-binding proteins					Collagenases				
cbp-1	pS2160-1p3	6780	9002	+	colA-1	pS2160-1p2	7213	7974	+
cbp-2	pS2160-1p2	26,836	29,052	+	colA-2	pS2160-1p2	342,610	343,371	+
cbp-3	Chromosome	2,703,734	2,703,862	−	Cell wall hydrolase				
					cwlJ	Chromosome	5,315,285	5,315,707	+

### Nano LC–MS/MS analysis of the whole proteome of Bt S2160-1

**Figure 3:**
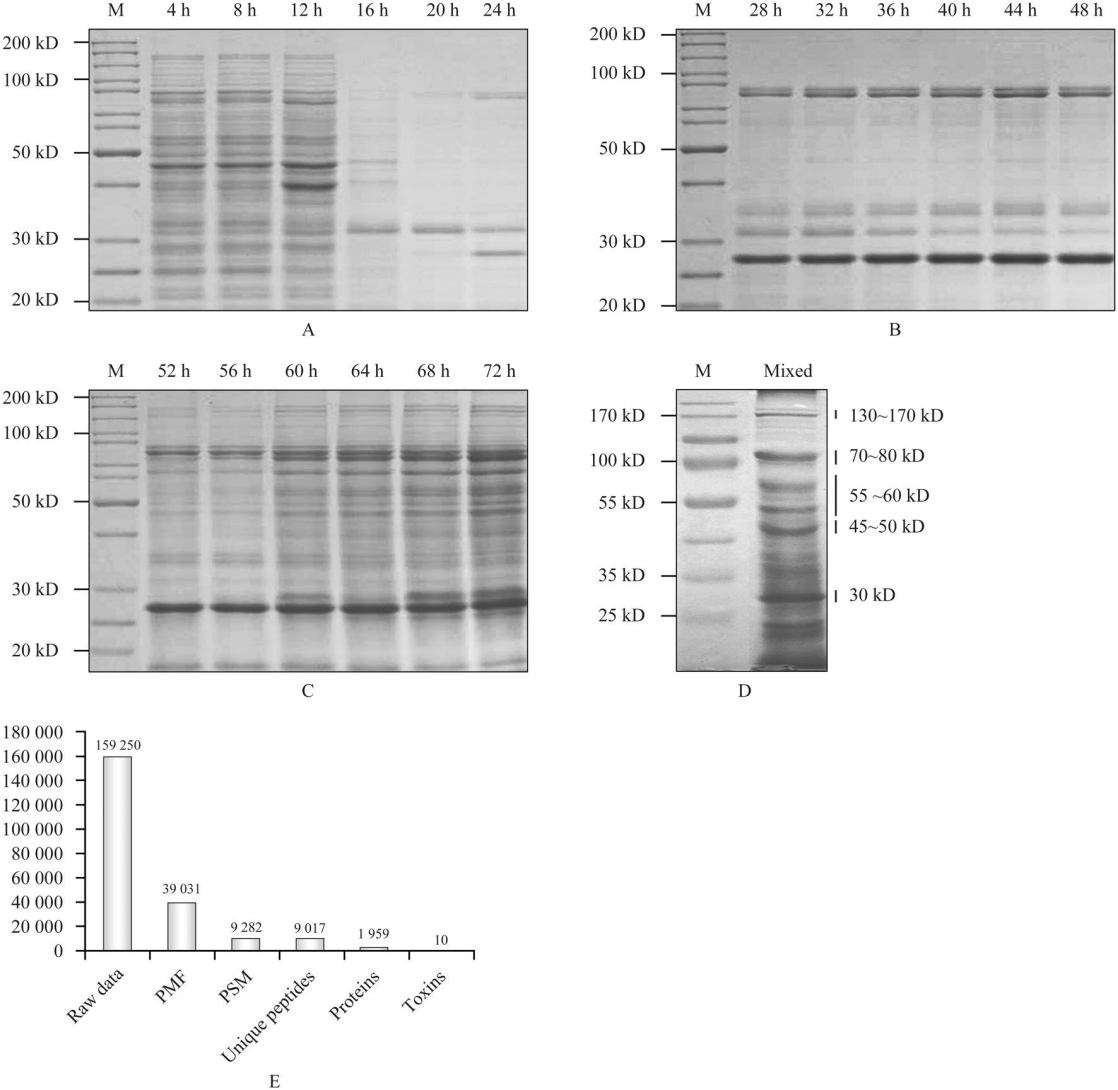
The expression profile of the proteins of Bt S2160-1 by SDS-PAGE analysis and whole proteome analysis of Bt S2160-1 by LTQ-Orbitrap Nano LC–MS/MS. *Note*: (**A**–**C**) SDS-PAGE of Bt S2160-1 proteins expressed over three consecutive days in which samples were collected once every 4 h at 18 time points covering 4–72 h; (**D**) the protein profile of Bt S2160-1 identified by SDS-PAGE based on mixing the expressed proteins harvested over three consecutive days; (**E**) Analysis of nano LC–MS/MS data based on a query of the SEQUEST database. M: Fermentas PageRuler™ Unstained Protein Ladder. *PMF* peptide mass fingerprinting, *PSM* peptide spectrum match.

The protein expression profile of strain Bt S2160-1 as determined by SDS-PAGE indicated that the toxin proteins exhibited a range of molecular masses, producing bands at ranges of 130–150 kDa, 70–80 kDa, 55–60 kDa, 45–50 kDa, and 30 kDa (Fig. 3A–D). The same band on an SDS-PAGE gel can be composed of several proteins of different molecular mass and protein expression can vary at different points in the life-cycle. Therefore, to try to identify as many expressed proteins as possible, all of the proteins expressed by the Bt S2160-1 strain from the vegetative phase of growth to late sporulation were analyzed by Nano LC–MS/MS.

As a result, 159,250 raw mass spectra data were obtained by LTQ-Orbitrap Elite MS analysis. Using the SEQUEST database, 39,031 PMFs (Peptide Mass Fingerprinting, PMF) and 9282 PSMs (Peptide Spectrum Match, PSM) were retrieved based on the predicted ORF protein data of the Bt S2160-1 genome and used as the target library. The target library was composed of 9017 unique peptides. Based on the obtained spectra, 1959 proteins with high scores were identified (Fig. 3E).

A total of 1959 proteins were identified in the MS analysis, representing a coverage of 29.89% (a total of 6553 encoded proteins in the genome sequence of Bt S2160-1). The remaining 70.11% of the predicted proteins were not identified in our analysis. The number of identified proteins in our study is greater than the number obtained by Huang et al.^34^, who identified a total of 1480 proteins of which 918, 703, and 778 were identified by LC–MS/MS from the middle vegetative, early sporulation, and late sporulation stages of growth, respectively.

Our higher number of identified proteins could be attributed to inclusion of a greater number of time points (18 sampling points) in the protein mixture that was analyzed. Therefore, apart from putative proteins predicted by the whole genome sequencing that may not be expressed as part of the growth of the strain, itself, other factors may be responsible for the incomplete number of proteins that were identified. First, the incomplete identification may have been due to the discontinuity in sampling, which may have excluded the collection of many proteins. Second, the methods used for sample preparation may have resulted in the loss of many proteins and/or the inability to obtain reliable spectra so that the proteins could not be identified. Lastly, the expression of some proteins may be so low that they did not produce spectra that could be used for identification.

10 of the 13 predicted toxin proteins were found to be expressed in our analysis of protein expression (Table 4). We identified ten of the toxin proteins encoded by these genes in our MS analysis of protein expression, however, three (pS2160-1p2_id_6113, pS2160-1p2_id_6159 and pS2160-1p2_id_6259) were not identified. These results indicate that the majority, if not all, of the predicted toxin proteins should be expressed. Failure to identify three of the encoded toxin proteins may be due to their absence in the collected samples, their level in the collected samples being too low to detect, or their loss during sample preparation. Inability to detect the presence of these three proteins does not mean they are not expressed in vivo. Therefore, subsequent in vivo and in vitro studies are needed to determine if these proteins are actually expressed and to determine their potential function.

**Table 4:** The predicted toxin proteins detected by LTQ-Orbitrap MS in total protein samples of Bt S2160-1.

Protein ID	Toxin	Score	Coverage	Proteins	Unique peptides	Peptides	PSMs	AAs	MW (kDa)	Calc. pI
pS2160-1p2_id_6063	Cry54Ba2	631.60	51.36	1	32	32	252	699	79.5	6.89
pS2160-1p2_id_6065	CryEa4	126.60	26.74	2	15	16	52	688	77.7	7.80
pS2160-1p2_id_6074	Cry69Aa-like	494.40	32.57	1	31	32	163	1216	137.2	5.82
pS2160-1p 2_id_6087	Cry50Ba2	324.41	81.85	1	27	27	119	658	73.8	5.74
pS2160-1p 2_id_6093	Cry4Ca1-like	474.49	36.6	1	29	29	149	1157	130.6	5.97
pS2160-1p2_id_6113*	Cry30Ga2	ND	ND	ND	ND	ND	ND	ND	ND	ND
pS2160-1p2_id_6137	Cry71Aa-like	44.83	30.65	2	8	9	22	697	79.3	8.21
pS2160-1p2_id_6145	Cry72Aa-like	14.51	17.2	1	4	4	8	692	77.8	7.36
pS2160-1p2_id_6159*	Cry70Aa-like	ND	ND	ND	ND	ND	ND	ND	ND	ND
pS2160-1p2_id_6167	Cyt1Da1-like	110.47	31.86	1	13	13	40	499	56.6	6.77
pS2160-1p2_id_6194	Vpb4Ca1-like	296.96	25.42	2	7	14	134	531	60.9	5.67
pS2160-1p 2_id_6259*	Cyt1Aa-like	ND	ND	ND	ND	ND	ND	ND	ND	ND
pS2160-1p 2_id_6293	Tpp80Aa1-like	11.83	9.65	1	3	3	5	373	42.2	6.55

## Discussion

At present, the whole genome sequencing has been widely used to mine insecticidal protein genes. Pacheco using whole genome sequencing analysis of *B. thuringiensis* GR007 reveals multiple pesticidal protein genes against *M. sexta* and *S. frugiperda* larvae ^35^. Naveenarani also using whole genome sequencing first reported *B. thuringiensis* (Bt 62) isolate harboring two novel *cry8* genes were toxic to *Holotrichia serrata*^36^. In the current study, the complete-genome of the mosquitocidal Bt S2160-1 strain was sequenced by MGISEQ-2000 platform and Oxford Nanopore Technologies (ONT) PromethION P24 device at BGI (Shenzhen, China) in 2021. Bt S2160-1 contains four mega-plasmids, pS2160-1p1, pS2160-1p2, pS2160-1p3 and pS2160-1p4, whose sequences were assembled with the help of bioinformatic software. The large plasmid pS2160-1p2 of Bt S2160-1 harbors all the genes that encode for potential pesticidal proteins (PPs), which are grouped in PAIs, namely PAI-1 and PAI-2 (Fig. 2). PAI-1 containing eleven PPs is 101.6 kb, while two genes encoding for PPs on PAI-2 is 30.6 kb (Fig. 2). Thus, it was apparent that all these toxin-like genes on plasmid pS2160-1p2 were clustered together.

Since Hacker et al^33^. first proposed the concept of pathogenicity islands in 1990, they have been the subject of much research. A pathogenicity island (PAI), also known as a virulence island, is a cluster of genes in pathogenic bacteria that have specific structural characteristics and functions, primarily coding for products related to pathogenic virulence and bacterial metabolism. Pathogenicity islands can be up to 190 kb in size, and are often located in or near a tRNA gene or on the flanks of insertion sequences^37^. Researchers have now identified numerous functionally-related clusters of genes, including those that encode antibiotics (antibiotic resistance islands) and clusters of genes that associated with adaptive metabolic properties (metabolic islands)^38^.

Pathogenicity islands are a set of virulence-related DNA sequences in pathogenic bacteria, whose presence may play a key role in the evolution of pathogens^34^. All potential proteins are encoded in large megaplasmids as PAIs with individual gene or grouped, accompanied by repeat sequences, insertion elements, and transposases, which may allow a higher recombination rate among diverse Bt strains^39,40^. Different Bt strains harbor several pesticidal proteins (PP), forming multiple PAI islands. The PAI-1.1 is the longest PAI region in Bt GR007 strain, containing seven different cry genes^35^, which are lepidopteran specific Cry proteins (Cry1Da, Cry1Id, Cry1Ja, Cry1Nb, Cry1Ab, Cry1Bb, and Cry1Hb)^41^. This PAI-1.1 also codifies for a cluster of five insecticidal toxin components (Tcs). The Tcs proteins were originally identified in enterobacteria *P. luminescens* and *Xenorhabdus nematophila*, which are symbiont of nematodes^42^. Here, the eleven predicted toxin genes (Cry54Ba, Cry30Ea4, Cry69Aa-like, Cry50Ba2-like, Cry4Ca1-like, Cry30Ga2, Cry71Aa-like, Cry72Aa-like, Cry70Aa-like, Cyt1Da2-like and Vpb4C1-like) located on PAI-1, which is speculated that the above Tcs proteins exist. PAI-2 contains two potential toxin genes (Cyt1Aa, and Tpp80Aa1) that are adjacent to each other, and Tpp80Aa1 (Cry80Aa1) is also recognized for its toxicity against Diptera insects *C. pipiens pallens*^23^. Otherwise, each predicted toxin gene is arranged on the plasmid as part of a gene cluster, and each predicted toxin gene is preceded or followed by one or more genes encoding transposases, such as IS4, IS6, IS1182, and Tn3 family transposases. There is also a sigma-family transcriptional regulator and an insertion sequence IS231S (transposable factor) downstream of all the toxin genes. Collectively, these elements form a mosquitocidal island similar to the virulence island in pathogenic bacteria.

For the next, we need to express all thirteen of the predicted toxic encoding genes in *Escherichia coli* and verify the expressed proteins by MALDI-TOF mass spectrometry. And subsequently bioassay will be performed to check the mosquitocidal activities for all these thirteen expressed proteins, even containing to determine synergistic activity of them against three-instar larvae of *C. pipiens*. Actually, we are just carrying out this experiment. But determining the exact contribution of each toxin (alone or in combination) to the overall toxicity of the crystal inclusions is difficult, even in the Bti strain whose synergistic activity between toxins has been well studied. Comparing the level of toxicity reported by different studies can also be problematic due to differences in experimental conditions. Factors that affect obtained levels of toxicity include host-dependent differences in the various expression systems used, differences in the size, quality, and solubility of crystal formation^43^; differences in protein solubility or in the form of the reprecipitated protein presented to the larvae, and differences in bioassay conditions (including larval age, dietary habits, larval batches, and natural variation in insect populations)^44^. Therefore, they will take us more time to determine all these mosquitocidal activities.

Previous studies have demonstrated that the IS231 insertion sequence, belonging to the IS4 family of transposases, may provide the mobility to cry genes that is needed to form typical composite transposons^33^. Notably, the flanking repeat sequence IS231W found in *B. thuringiensis* subsp. *israelensis* (Bti) is adjacent to the *cry11Aa* gene^45^. Similarly, the flanking repeat sequences IS231S and IS132C are also found in both pS2160-1p1 and pS2160-1p2 plasmids, and all the 13 toxin genes located on the pS2160-1p2 plasmid cluster together (Fig. 2). Therefore, we speculate that the cluster of toxin-encoding genes in the Bt S2160-1 strain represent a mosquitocidal toxin island. Importantly, however, several IS231-family insertion sequences associated with the cry gene structure are also observed in the pS2160-1p2 plasmid. These elements may provide the mobility needed for cry genes to form a typical composite transposon^46,47^.

A whole-proteomic analysis provided evidence that ten of the thirteen predicted toxic proteins were expressed during the growth phases of the Bt S2160-1 strain, however, three of the predicted toxic proteins (pS2160-1p2_id_6113, pS2160-1p2_id_6159 and pS2160-1p2_id_6259) of the whole proteome could not be confirmed in nano LC–MS/MS analysis. Due to the complicated sample preparation of proteomic analysis, multiple recovery is required, and after these operations, the three proteins in sampling were too low to be almost absent for detecting. In future studies, we can increase the amount of initial protein samples and reduce the steps of protein loss at the same time, or setting several parallel protein treatments, reducing the amount of protein loss after various treatments, mix evenly to ensure all proteins can reach the detected content. When RT-PCR was conducted with Bt S2160-1 strain cDNA as template, however, transcription products for these three genes were obtained (data not shown), indicating that these three genes have transcripts. Therefore, we will change alternative detection methods for these three predicted toxins.

Otherwise, the mechanisms of sporulation and ICP formation in *B. thuringiensis* have been investigated for many years^48–51^, the signaling pathways involved in sporulation and ICP formation was reported little, while Zheng et al.^52^ found that CdaS promotes sporulation in *B. thuringiensis*, some c-di-AMP targets were obtained by affinity method, which are the effector proteins that affect sporulation and the formation of parasporal crystals. When exposed to susceptible *G. mellonella*, *B. thuringiensis* rapidly modulated gene expression to affect sporulation. Therefore, it is very interesting for us to figure out the how these 13 ICP encoding genes formed and what’s the relationship among these toxins with sporulation in Bt S2160-1 strain.

Overall, the present study provided a complete-genome assembly of Bt S2160-1 as an alternative to the use of Bti and identified the presence of a dominant pathogenicity island of Bt toxins. In addition, for the next step, we need to clone, express and determine the mosquitocidal activity of all these 13 toxic proteins against mosquitoes, and to check whether synergistic effect existed in these toxic proteins generating high efficiency mosquitocidal activity of Bt S2160-1 strain. Therefore, the use of Bt S2160-1 to control mosquitoes, as well as other dipteran pests, would provide a great benefit to human health and agriculture.

## Conclusion

In summary, the present study identified the existence of two mosquitocidal toxin islands in Bt S2160-1 strain containing thirteen potential toxic genes, and ten of thirteen predicted genes were confirmed by whole-proteome analysis using LTQ-Orbitrap LC–MS/MS, which will provide important genomic information for understanding the insecticidal mechanism of *B. thuringiensis*.

## Data Availability

The datasets used and/or analyzed during the current study are available from the corresponding author on reasonable request.

## References

[1] Schnepf E (1998). *Bacillus thuringiensis* and its pesticidal crystal proteins. Microbiol. Mol. Biol. Rev..

[2] de Maagd RA, Bravo A, Crickmore N (2001). How *Bacillus thuringiensis* has evolved specific toxins to colonize the insect world. Trends Genet..

[3] Margalith Y, Ben-Dov E, Rechcigl J, Rechcigl N (2000). Biological control by *Bacillus thuringiensis* subsp. *israelensis*. Insect Pest Management: Techniques for Environmental Protection.

[4] Fillinger U, Lindsay SW (2006). Suppression of exposure to malaria vectors by an order of magnitude using microbial larvicides in rural Kenya. Trop. Med. Int. Health.

[5] Carlton BC, Gonzalez JM, Hoch JA, Setlow P (1985). Plasmids and delta-endotoxin production in different subspecies of *Bacillus thuringiensis*. Molecular Biology of Microbial Differentiation.

[6] Sekar V, de Barjac H, Sutherland DJ (1990). Genetics of *Bacillus thuringiensis israelensis*. Bacterial Control of Mosquitoes and Lack Flies.

[7] Ben-Dov E, Einav M, Peleg N, Boussiba S, Zaritsky A (1996). Restriction map of the 125-kilobase of *Bacillus thuringiensis* subsp. *israelensis* carrying the genes that encode delta-endotoxins active against mosquito larvae. Appl. Environ. Microbiol..

[8] Ben-Dov E, Nissan G, Peleg N, Manasherob R, Boussiba S, Zaritsky A (1999). Refined, circular restriction map of the *Bacillus thuringiensis* subsp. *israelensis* plasmid carrying the mosquito larvicidal genes. Plasmid.

[9] Berry C (2002). Complete sequence and organization of pBtoxis, the toxin-coding plasmid of *Bacillus thuringiensis* subsp. *israelensis*. Appl. Environ. Microbiol..

[10] Delécluse A, Rosso ML, Ragni A (1995). Cloning and expression of a novel toxin gene from *Bacillus thuringiensis* subsp. *jegathesan* encoding a highly mosquitocidal protein. Appl. Environ. Microbiol..

[11] Orduz S, Realpe M, Arango R, Murillo LA, Delécluse A (1998). Sequence of the cry11Bb1 gene from *Bacillus thuringiensis* subsp. *medellin* and toxicity analysis of its encoded protein. Biochim. Biophys. Acta.

[12] Rosso ML, Delécluse A (1997). Contribution of the 65-kilodalton protein encoded by the cloned gene cry19A to the mosquitocidal activity of *Bacillus thuringiensis* subsp. *jegathesan*. Appl. Environ. Microbiol..

[13] Abdullah MAF, Dean DH (2004). Enhancement of Cry19Aa mosquitocidal activity against *Aedes aegypti* by mutations in the putative loop regions of domain II. Appl. Environ. Microbiol..

[14] Hwang SH, Saitoh H, Mizuki E, Higuchi K, Ohba M (1998). A novel class of mosquitocidal delta-endotoxin, Cry19B, encoded by a *Bacillus thuringiensis* serovar higo gene. Syst. Appl. Microbiol..

[15] Valtierra-de-Luis D, Villanueva M, Berry C, Caballero P (2020). Potential for *Bacillus thuringiensis* and other bacterial toxins as biological control agents to combat dipteran pests of medical and agronomic importance. Toxins (Basel).

[16] Sun Y, Zhao Q, Xia L, Ding X, Hu Q, Federici BA (2013). Identification and characterization of three previously undescribed crystal proteins from *Bacillus thuringiensis* subsp. *Jegathesan*. Appl. Environ. Microbiol..

[17] Saitoh H, Hwang SH, Park YS, Higuchi K, Mizuki E, Ohba M (2000). Cloning and characterization of a *Bacillus thuringiensis* serovar higo gene encoding a novel class of the delta-endotoxin protein, Cry27A, specifically active on the *Anopheles* mosquito. Syst. Appl. Microbiol..

[18] Lee HK, Gill SS (1997). Molecular cloning and characterization of a novel mosquitocidal protein gene from *Bacillus thuringiensis* subsp. *fukuokaensis*. Appl. Environ. Microbiol..

[19] Roh JY (2009). *Bacillus thuringiensis* serovar mogi (flagellar serotype 3a3b3d), a novel serogroup with a mosquitocidal activity. J. Invertebr. Pathol..

[20] Ito T, Ikeya T, Sahara K, Bando H, Asano S (2006). Cloning and expression of two crystal protein genes, cry30Ba1 and cry44Aa1, obtained from a highly mosquitocidal strain, *Bacillus thuringiensis* subsp. entomocidus INA288. Appl. Environ. Microbiol..

[21] Soares-da-Silva J (2017). Molecular characterization of the gene profile of *Bacillus thuringiensis* Berliner isolated from Brazilian ecosystems and showing pathogenic activity against mosquito larvae of medical importance. Acta Trop..

[22] Juárez-Pérez V, Porcar M, Orduz S, Delécluse A (2003). Cry29A and Cry30A: Two novel δ-endotoxins isolated from *Bacillus thuringiensis serovar medellin*. Syst. Appl. Microbiol..

[23] Zhou Y, Wu Z, Zhang J, Wan Y, Jin W, Li Y, Fang X (2020). Cry80Aa1, a novel *Bacillus thuringiensis* toxin with mosquitocidal activity to *Culex pipiens pallens*. J. Invertebr. Pathol..

[24] Bravo A, Gill SS, Soberón M (2005). Bacillus thuringiensis Mechanisms and Use.

[25] Zhou Y, Wu Z, Zhang J, Wan Y, Jin W, Li Y, Fang X (2020). *Bacillus thuringiensis* novel toxin Epp is toxic to mosquitoes and prodenia litura larvae. Braz. J. Microbiol..

[26] Zhang W (2012). Characterization of a new highly mosquitocidal isolate of *Bacillus thuringiensis*—An alternative to *Bti*?. J. Invertebr. Pathol..

[27] Zhang W (2014). Identification of a mosquitocidal toxin from *Bacillus thuringiensis* using mass spectrometry. World J. Microbiol. Biotechnol..

[28] Zhang W (2017). Characterization of a novel mosquitocidal toxin of Cry50Ba and its potential synergism with other mosquitocial toxins. Toxicon.

[29] Delcher AL, Bratke KA, Powers EC, Salzberg SL (2007). Identifying bacterial genes and endosymbiont DNA with Glimmer. Bioinformatics.

[30] Borodovsky M, Mills R, Besemer J, Lomsadze A (2003). Prokaryotic gene prediction using GeneMark and GeneMark.hmm. Curr. Protoc. Bioinform..

[31] Zheng Z (2020). The CRISPRCas systems were selectively inactivated during evolution of *Bacillus cereus* group for adaptation to diverse environments. ISME J..

[32] Crickmore N, Berry C, Panneerselvam S, Mishra R, Connor TR, Bonning BC (2021). A structure-based nomenclature for *Bacillus thuringiensis* and other bacteria-derived pesticidal proteins. J. Invertebr. Pathol..

[33] Hacker J (1990). Deletions of chromosomal regions coding for fimbriae and hemolysins occur in vitro and in vivo in various extraintestinal *Escherichia coli* isolates. Microb. Pathog..

[34] Huang SY (2012). Proteomic analysis of *Bacillus thuringiensis* at different growth phases by using an automated online two-dimensional liquid chromatography–tandem mass spectrometry strategy. Appl. Environ. Microbiol..

[35] Pacheco S, Gómez I, Chiñas M, Sánchez J, Soberón M, Bravo A (2021). Whole genome sequencing analysis of *Bacillus thuringiensis* GR007 reveals multiple pesticidal protein genes. Front. Microbiol..

[36] Naveenarani M, Suresha GS, Srikanth J, Hari K, Sankaranarayanan C, Mahesh P, Nirmala R, Swathik CP, Crickmore N, Ram B, Appunu C, Singaravelu B (2022). Whole genome analysis and functional characterization of a novel *Bacillus thuringiensis* (Bt 62) isolate against sugarcane white grub *Holotrichia serrata* (F). Genomics.

[37] SunSullivan JT, Ronson CW (1998). Evolution of rhizobia by acquisition of a 500-kb symbiosis island that integrates into a phe-tRNA gene. Proc. Natl. Acad. Sci. U.S.A..

[38] Che DS, Hasan MS, Chen B (2014). Identifying pathogenicity islands in bacterial pathogenomics using computational apmmproaches. Pathogens.

[39] Mahillon J, Chandler M (1998). Insertion sequences. Microbiol. Mol. Biol. Rev..

[40] Fiedoruk K, Daniluk T, Mahillon J, Leszczynska K, Swiecicka I (2017). Genetic environment of cry1 genes indicates their common origin. Genome Biol. Evol..

[41] Crickmore N, Berry C, Panneerselvam S, Mishra R, Connor TR, Bonning BC (2020). A structure-based nomenclature for *Bacillus thuringiensis* and other bacteria-derived pesticidal proteins. J. Invertebr. Pathol..

[42] Waterfield NR, Bowen DJ, Fetherstone JD, Perry RD, Hffrench-Constant RH (2001). The tc genes of *Photorhabdus*: A growing family. Trends Microbiol..

[43] Crickmore N, Bone EJ, Williams JA, Ellar DJ (1995). Contribution of the individual components of the d-endotoxin crystal to the mosquitocidal activity of *Bacillus thuringiensis* subsp. *israelensis*. FEMS Microbiol. Lett..

[44] Robertson JL, Preisler HK, Ng SS, Hickle LA, Gelernter WD (1995). Natural variation: A complicating factor in bioassays with chemical and microbial pesticides. J. Econ. Entomol..

[45] Mahillon J, Seurinck J, van Rompuy L, Delcour J, Zabeau M (1985). Nucleotide sequence and structuralorganization of an insertion sequence element (IS231) from *Bacillus thuringiensis* strain berliner 1715. EMBO J..

[46] Hallet B, Rezsöhazy R, Delcour J (1991). IS231A from *Bacillus thuringiensis* is functional in *Escherichia coli*: Transposition and insertion specificity. J. Bacteriol..

[47] OrduzMenou G, Mahillon J, Lecadet MM, Lereclus D (1990). Structural and genetic organization of IS232, a new insertion sequence of *Bacillus thuringiensis*. J. Bacteriol..

[48] Baum JA, Malvar T (1995). Regulation of insecticidal crystal protein production in *Bacillus thuringiensis*. Mol. Microbiol..

[49] Chang L, Grant R, Aronson A (2001). Regulation of the packaging of *Bacillus thuringiensis* delta-endotoxins into inclusions. Appl. Environ. Microbiol..

[50] Gong Y (2012). Comparative proteomic analysis revealed metabolic changes and the translational regulation of Cry protein synthesis in *Bacillus thuringiensis*. J. Proteom..

[51] Wang J (2013). The metabolic regulation of sporulation and parasporal crystal formation in *Bacillus thuringiensis* revealed by transcriptomics and proteomics. Mol. Cell Proteom..

[52] Zheng C, Ma Y, Wang X, Xie Y, Ali MK, He J (2015). Functional analysis of the sporulation-specific diadenylate cyclase CdaS in *Bacillus thuringiensis*. Front. Microbiol..

